# Editorial: Cognition Across the Psychiatric Disorder Spectrum: From Mental Health to Clinical Diagnosis

**DOI:** 10.3389/fpsyt.2015.00110

**Published:** 2015-08-04

**Authors:** Caroline Gurvich, Susan L. Rossell

**Affiliations:** ^1^Monash Alfred Psychiatry Research Centre (MAPrc), The Alfred Hospital, Central Clinical School, Monash University, Melbourne, VIC, Australia; ^2^Faculty of Health, Arts and Design, Brain and Psychological Sciences Research Centre, Swinburne University of Technology, Melbourne, VIC, Australia

**Keywords:** schizotypy, schizophrenia, cognition, neurocognition, psychopathology, schizophrenia spectrum

Schizophrenia is a common psychiatric diagnosis affecting approximately 0.7% of the population worldwide (1). Cognitive symptoms in schizophrenia, such as impaired memory, poor attention/information processing, and difficulties with executive functions, are a core feature of schizophrenia and strongly related to quality of life and functional outcomes, yet generally respond poorly to current treatment options (2, 3). Further research exploring the basis of cognitive impairments in schizophrenia is essential to allow for better targeted treatment options. Improved cognition would pave a much better path to functional recovery for people with schizophrenia, for example, increasing the chances of someone being able to return to work or study when their positive psychotic symptoms are stabilized. One avenue that has emerged as a way to study symptoms of schizophrenia, such as cognition, is the study of schizotypy. Schizotypy refers to subclinical psychotic experiences (which can include odd behaviors, strange speech, unusual perceptual experiences, and social anhedonia) that are distributed along a continuum, from mental health to a diagnosable psychiatric disorder. While the term schizotypy was coined over six decades ago (4), research examining schizotypal traits in non-clinical populations has rapidly expanded over the last few years (a recent PsychInfo search of the term “schizotypy” – conducted 19/05/2015 – showed more than 5850 publications, with more than half of those publications in the last 5 years). The exploration of schizotypy may help elucidate many factors related to the etiology and development of schizophrenia spectrum psychopathology, including cognition. It is timely and important to collate current research exploring schizotypy and determine how this avenue of research can inform our understanding of cognition across the schizophrenia spectrum.

The aim of this Research Topic is to provide updated knowledge, reviews, and opinion pieces in relation to cognition, schizotypy, and the schizophrenia continuum. Drawing on a variety of perspectives and collating the results of several experimental studies will inform on the current status of schizotypy research and allow future research directions to be identified. Three key sections will be explored: schizotypy as a construct, including theoretical and methodological considerations when assessing schizotypy; a comprehensive review of dopaminergic contributions to schizotypy; and, several empirical research studies exploring cognition and symptomatology across the schizophrenia and schizotypy spectrum.

Schizotypy has been conceptualized as both taxonic/categorical and dimensional. The categorical approach (5) is based on a disease model of mental illness and considers schizotypy to be a subclinical expression of the symptoms of schizophrenia that are present in a small subgroup of the population (approximately 10%). The fully dimensional approach (6) stems from Eysenck’s dimensional views of personality and describes schizotypy as continuous throughout the general population with higher levels of schizotypy, in combination with other etiological risk factors, to indicate a greater risk for developing schizophrenia. In this Research Topic, Mason (7) provides an interesting opinion piece that acknowledges that while there are theoretical differences between these models, both dimensional and categorical approaches may have validity and research utility in relation to schizotypy.

While the study of schizotypy is interesting in its own right (for example, as a dimension of personality), schizotypy also offers a number of advantages for studying schizophrenia liability. While many of the confounding factors associated with schizophrenia, such as hospitalization, social isolation, medication/illicit drug use, and health complications, can be controlled when using a non-clinical schizotypy population, there remain extraneous factors that should be considered when schizotypy samples are employed. Neill (8), in this Research Topic, considers some of these methodological issues, such as age, education, relative status, abuse history, and religion, when recruiting and analyzing schizotypy samples. As Neill concludes, schizotypy research is rapidly expanding and it is critical that as this field moves forward, the many potential influences on schizotypy are considered to ensure studies are well designed and statistically valid.

There is considerable evidence indicating an overlap between schizotypy and schizophrenia in relation to behavioral, cognitive, brain structure, and brain function measures. At a neurochemical level, there is a long-standing literature linking dopamine to the pathophysiology of schizophrenia (9, 10) and an accumulating literature indicating a role for dopamine in schizotypy. In this Research Topic, Mohr and Ettinger (11) review the association between dopamine, schizotypy, and cognition across a wide range of methods, including experimental pharmacological challenge studies, dopamine-sensitive cognitive and behavioral measures, molecular studies of genes that involve dopamine transmission, and molecular imaging studies of the dopamine system. The authors conclude that there is some evidence of an association between altered dopamine neurotransmission and schizotypy, particularly positive schizotypy. Importantly, the authors provide suggestions for future avenues of research that will inform neurobiological and cognitive models of the schizophrenia spectrum, paving the way for potential neuropharmacological treatments.

The second key component of this Research Topic presents several empirical studies exploring different aspects of cognition and symptomatology across the schizophrenia/schizotypy continuum.

There is a long history of experimental paradigms that attempt to induce psychotic-like experiences (such as perceptual disturbances, paranoia, and anhedonia), many of these involving various means of sensory deprivation. The effects of brief sensory deprivation and the associated experience of psychotic-like experiences are explored in this Research Topic by Daniel et al. (12) in relation to schizotypal traits (high- vs. low-hallucination proneness). The study findings indicate that sensory deprivation can be a useful, non-pharmacological tool for temporarily inducing psychotic-like states across all individuals, with schizotypal traits relating to greater levels of perceptual distortions.

The multidimensionality of schizotypy is addressed in several studies in this Research Topic. Ford and Crewther (13) explore shared phenotypes across the autism and schizophrenia spectrum disorders. They conducted a factor analysis on items from the autism spectrum quotient (AQ) and the schizotypal personality questionnaire (SPQ) in a non-clinical population. Results revealed a social disorganization phenotype common to both schizotypy and autism, as well as factors specific to both spectrums of personality.

Two studies are presented that explore the link between schizotypy and neurocognition. Louise et al. (14) investigate neurocognition and schizotypy subtypes or factors using the Oxford-Liverpool inventory of feelings and experiences (O-LIFE) in an adult community sample that accounted for psychiatric illness and family history; and hence, allowing for the exploration of both cognitive functioning and potential compensatory mechanisms in individuals who have passed the peak onset times for developing schizophrenia. Results indicated a positive relationship between poorer inhibitory control and the schizotypy factors of positive schizotypy, cognitive disorganization, and impulsive non-conformity, as well as a positive relationship between negative schizotypy and poorer attention/processing speed and reasoning and problem-solving capacity. Herzig et al. (15), in this Research Topic, explore neurocognition and schizotypy, with their primary focus being how substance use attenuates cognition. In their university-aged sample, Herzig et al. found a trend toward higher positive schizotypy scores in their “cannabis users” group (as compared to the non-cannabis users). In relation to the three cognitive tasks that they assessed (verbal short-term recall, trail-making task, and two-back working memory task), they failed to find a relationship between schizotypy scores and cognition, but found enhanced cannabis use predicted decreased verbal short-term memory, whereas enhanced alcohol use predicted reduced working memory performance. These results highlight the potential importance of controlling for substance use when exploring the links between schizotypy and cognition.

An interesting study assessing the integrity of body representations in individuals with schizophrenia is presented by Graham et al. (16), with their focus on passivity symptoms (i.e., the belief that one’s thoughts or actions are controlled by an external agent). Their results highlight self-abnormalities in schizophrenia and provide evidence for both stable trait abnormalities and state changes that depend on passivity symptom profiles. Batty et al. (17) explore neurophysiological correlates of face processing in schizotypy. Their results suggest that high schizotypes (as measured by the cognitive disorganization factor of the O-LIFE) demonstrate neurophysiological anomalies relating to the early, configural stages of face processing (N170 component), a finding that has been demonstrated in schizophrenia samples. As the authors discuss, the high schizotypal group demonstrated intact behavioral performance indicating anomalies in neural processes during the earlier stages of face processing are possibly corrected during the later stages of processing.

Within the framework of the dimensional model of schizotypy, high levels of schizotypy are not necessarily pathological but possibly beneficial, particularly in relation to positive schizotypy (18). For example, there is much research positing a link between high schizotypy and the socially valued cognitive attribute of creativity [e.g., Ref. (18, 19)]. In this Research Topic, Grant et al. (20) explore the interactions between positive schizotypy and verbal intelligence in relation to stimulus ambiguity and false-positive errors. The findings from Grant et al.’s study indicate that both state and trait positive schizotypy explain much of the variance in the production of false-positive errors/stimulus ambiguity (or by inference, hallucinatory experiences) and verbal intelligence moderates the relationship between schizotypy and the production of false-positive perceptions of ambiguous stimuli.

In relation to potential interventions across the psychosis spectrum, Lim and Gleeson (21), in this Research Topic, explore the link between loneliness and the psychosis spectrum. The authors highlight the growing interest in the relationship between loneliness and mental health disorders; and note that the more specific, and crucial, relationship between loneliness and psychotic disorders has been overlooked. A well-designed intervention to target loneliness for individuals with psychosis is warranted and may even reduce the risk of developing psychosis or experiencing a relapse of psychotic symptoms. Lim and Gleeson provide helpful guidelines to move this area of research forward.

To conclude, the articles in this Research Topic represent an interesting array of opinions, reviews, and empirical studies that contribute to the study of schizotypy. Collectively, they highlight the heuristic potential of the study of schizotypy, particularly in relation to better understanding cognition across the schizophrenia spectrum.

## Conflict of Interest Statement

The authors declare that the research was conducted in the absence of any commercial or financial relationships that could be construed as a potential conflict of interest.
